# Phase Shift Effects in Fabry-Perot Interferometry

**DOI:** 10.6028/jres.064A.020

**Published:** 1960-06-01

**Authors:** Charles J. Koester

## Abstract

A method is demonstrated for utilizing in Fabry-Perot interferometry the data on reflection phase shift dispersion obtained from fringes of equal chromatic order. Unknown wavelengths can be calculated from the Fabry-Perot patterns obtained with a large etalon spacing, even without prior knowledge of the phase shift of the reflecting surfaces. When the theoretical phase shift as a function of wavelength is known approximately, then the correct orders of interference can be determined for both the Fabry-Perot fringes and fringes of equal chromatic order. From the wavelengths of the latter the phase shift dispersion can be measured to an accuracy of about 10 A. The method is especially useful for reflectors with large dispersion of phase shift, such as multilayers. Results in the visible spectrum are reported for aluminum films and a pair of dielectric 15-layer broadband reflectors.

## 1. Introduction

In the field of interferometric length and wavelength measurement it has long been recognized that the dispersion of phase shift on reflection must be considered. In Fabry-Perot interferometry, procedures for determining and/or correcting for phase shift dispersion have been described by Fabry and Buisson [1],^2^ Eversheim [2], Meggers [3], Bauer [4], Jackson [5], Meissner [6], Barrell and Teasdale-Buckell [7], and by Rank and Bennett [8].

In wavelength intercomparison, the phase shift dispersion enters the calculations at two points: (1) The determination of the etalon spacing, and (2) the calculation of the unknown wavelength.

The purpose of this paper is to describe how the accurate measurement of phase shift dispersion by fringes of equal chromatic order (*feco*) [9, 10, 11] can be used with Fabry-Perot interferometry in the determination of the etalon spacing and in precise measurement of unknown wavelengths.

### 1.1. Determination of Etalon Spacing

The method of exact fractions [12, 13] is generally used to determine the etalon spacing from the observed interference pattern. For three known wavelengths, λ*_a_*, λ*_b_*, λ*_c_*, the fractional orders *f_a_*, *f_b_*, and *f_c_*, are calculated [6, 14] from the diameters of the circular fringes. Then the integral order numbers, *m_a_*, *m_b_*, *m_c_*, and the exact etalon spacing, *t*, are found by trying many different integers for *m_a_, m_b_*, and *m_c_* until the products (*m_a_+f_a_*)λ*_a_*, (*m_b_*+*f_b_*)λ*_b_*, and (*m_c_*+*f_c_*)λ*_c_* are all essentially equal. The etalon spacing is then set equal to

12(ma+fa)λa=12(mb+fb)λb=12(mc+fc)λc. (1)

This method obviously ignores the phase shift. The quantity ½(*m*+*f*)λ really represents the optical spacing of the etalon plates, which is the physical separation plus the phase shift on reflection for the particular wavelength. Since in general the phase shift is a function of wavelength, the quantity ½(*m+f*)λ is not constant as implied in eq (1) but is a function of wavelength. It is fortunate that for the metals aluminum and silver which are commonly used on Fabry-Perot mirrors, the phase shift dispersion is such that it is possible to find integers *m_a_*, *m_b_*, and *m_c_* which satisfy eq (1). When two different etalon spacings are determined by this method correct wavelengths values can be calculated.

Multilayer reflectors [15, 16, 17, 18] are finding considerable application in interferometry because of their low absorbance and high reflectance. For the common type of high reflection coating consisting of alternate high and low index layers, each of quarter-wave thickness for wavelength λ, the phase shift dispersion is small in the wavelength region near λ. Outside this spectral region the phase shift dispersion is much larger than for silver and aluminum mirrors. A 15-layer broadband high reflectance multilayer described by Baumeister and Stone [19] has a very large phase shift dispersion [20]. In such cases of high phase shift dispersion it is possible that integers cannot be found which will satisfy eq (1), or wrong integers may be selected.

Rank and Bennett [8] have described a method for using calculated values of the phase shift to correct the order number of interference in a Fabry-Perot etalon. Baumeister and Jenkins [20] have described another method for employing calculated phase shifts and have applied it to a nine-layer coating. The latter authors have also made measurements of the phase shift produced by the 15-layer broadband multilayer using Fizeau fringes [1]. The precision of this method was limited by the broad two-beam fringes used for comparison.

### 1.2. Calculation of the Unknown Wavelength

For work of highest accuracy, the above method of exact fractions is used only to determine the correct order numbers. The etalon spacing and the unknown wavelength are computed from a standard wavelength λ*_s_* as follows. The method eliminates phase shift dispersion errors.

The fractional orders of interference, *f_x_* and *f_s_* are obtained for the unknown wavelength, λ*_x_*, and for the standard wavelength, which can be one of the wavelengths used to determine the etalon spacing. The equations for interference at normal incidence are

$$\left(m_{x}+f_{x})\lambda_{x}=2t+2\phi (\lambda_{x}\right)$$ (2)

and

$$(m_{s}+f_{s})\lambda_{s}=2t+2\phi (\lambda_{s}).$$ (3)

Here *ϕ*(λ) is the phase shift in units of length; it is the additional optical path introduced on reflection. There are as many as four unknowns in these equations: λ*_x_, t*, *ϕ*(λ*_x_*), and *ϕ*(λ*_s_*). (The unknown wavelength, λ*_x_*, must be known in advance with sufficient accuracy to determine the order number, *m_x_*.)

If another etalon spacing, *t′*, is used, two more equations are obtained

$$(m_{x}^{\prime }+f_{x}^{\prime })\lambda_{x}=2t^{\prime }+2\phi (\lambda_{x}),$$ (4)

$$(m_{s}^{\prime }+f_{s}^{\prime })\lambda_{s}=2t^{\prime }+2\phi (\lambda_{s}).$$ (5)

The order number $m_{s}^{\prime }$ can be determined by the method of exact fractions described above. Again $m_{x}^{\prime }$ is determined from the approximate value of λ*_x_*. The four equations can be solved [6] for λ*_x_* by forming the difference between the two equations involving λ*_x_*, also between the two equations involving λ*_s_*, and equating the differences.

$$\lambda_{x}=\lambda_{s}\frac{m_{s}+f_{s}-m_{s}^{\prime }-f_{s}^{\prime }}{m_{x}+f_{x}-m_{x}^{\prime }-f_{x}^{\prime }}.$$ (6)

The phase shift terms are thereby eliminated. The difference in etalon spacing, *t−t′*, is obtained by subtracting eq (5) from eq (3)

$$t-t^{\prime }=\frac{1}{2}\lambda_{s}(m_{s}+f_{s}-m_{s}^{\prime }-f_{s}^{\prime }).$$

Variations of the above method of analysis have been used by Meggers [3], Jackson [5], and Meissner [6]. The variations make analysis of an extended series of data more convenient, but fundamentally they depend on the measured quantities, *f_s_*, $f_{s}^{\prime }$, *f_x_*, and $f_{x}^{\prime }$ in the manner shown in eq (6).

The order numbers *m_s_*, $m_{s}^{\prime }$, *m_x_*, and $m_{x}^{\prime }$ are integers which can be determined without ambiguity in cases where *ϕ*(λ*_s_*) and *ϕ*(λ*_x_*) can be adequately approximated. The accuracy with which λ*_x_* can be calculated from eq (6) therefore depends on the accuracy with which the fractions, *f*, can be measured. In particular, the error in λ*_x_* is given approximately by

$${\left(\frac{\Delta \lambda_{x}}{\lambda_{x}}\right)}^{2}={\left(\frac{\Delta f_{s}}{m_{s}-m_{s}^{\prime }}\right)}^{2}+{\left(\frac{\Delta f_{s}^{\prime }}{m_{s}-m_{s}^{\prime }}\right)}^{2}+{\left(\frac{\Delta f_{x}}{m_{x}-m_{x}^{\prime }}\right)}^{2}+{\left(\frac{\Delta f_{x}^{\prime }}{m_{x}-m_{x}^{\prime }}\right)}^{2}$$ (7)

where Δλ_x_, Δ*f*_s_, $\Delta f_{s}^{\prime }$, Δf_x_, and $\Delta f_{x}^{\prime }$ represent the standard deviations in the values of λ_x_, *f*_s_, $f_{s}^{\prime }$, *f*_x_, and $f_{x}^{\prime }$, respectively.

If the phase shift dispersion can be determined by independent measurements, then the unknown wavelength can be calculated from eqs (2) and (3)

$$\lambda_{x}=\frac{\left(m_{s}+f_{s})\lambda_{s}+2\phi (\lambda_{x})-2\phi (\lambda_{s}\right)}{m_{x}+f_{x}}.$$ (8)

The error in λ*_x_* is given approximately by

$${\left(\frac{\Delta \lambda_{x}}{\lambda_{x}}\right)}^{2}={\left(\frac{\Delta f_{s}}{m_{s}}\right)}^{2}+{\left(\frac{\Delta f_{x}}{m_{x}}\right)}^{2}+{\left(\frac{2\Delta \phi (\lambda_{x})}{m_{s}\lambda_{s}}\right)}^{2}+{\left(\frac{2\Delta \phi (\lambda_{s})}{m_{s}\lambda_{s}}\right)}^{2}.$$ (9)

Comparison of eqs (7) and (9) shows under what conditions the second method (eq 8) gives more accurate results than the first method (eq 6). For a given value of Δ*f_s_*, the value of Δ*f_s_*/*m_s_* in eq (9) will be smaller than $\Delta f_{s}/(m_{s}-m_{s}^{\prime })$ in eq (7). However, the difference need not be great if $m_{s}^{\prime }$ is small compared to *m_s_* (that is, if the smaller etalon spacing, *t′*, is small compared to the large etalon spacing, *t*).

The greatest gain is realized if 2Δ*ϕ*(λ*_s_*)/*m_s_*λ*_s_* can be made significantly smaller than $\Delta f_{s}^{\prime }/(m_{s}-m_{s}^{\prime })$, similarly for the quantities involving the unknown wavelength, λ*_x_*. Since it is possible to have $m_{s}-m_{s}^{\prime }\approx m_{s}$, this requirement can be written as 2Δ*ϕ*/λ<Δ*f*′. The limit on determination of the fraction *f*′ from the diameters of the circular fringes is about Δ*f*′= 0.01 [21,22]. It will be shown that when fringes of equal chromatic order are used to determine the phase shift dispersion, the quantity 2Δ*ϕ*/λ can be considerably less than 0.01.

## 2. Theoretical Calculation of Phase Shift on Reflection

**Figure 1:**
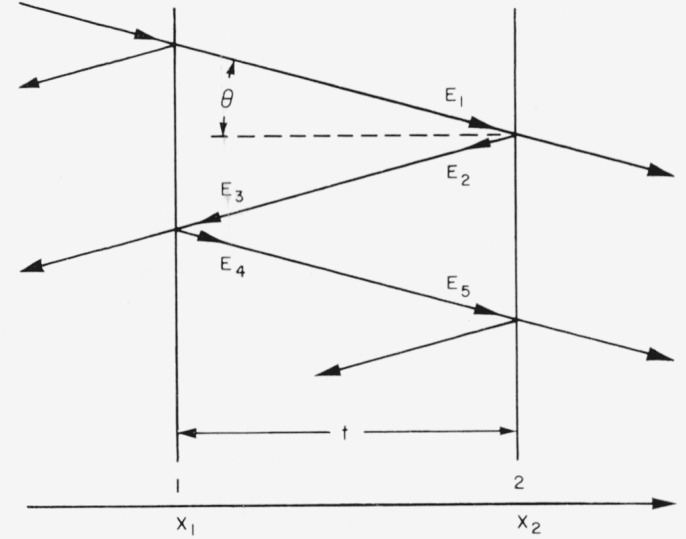
Schematic diagram of interferometer. The reflecting surfaces are denoted by 1 and 2. For *feco* and for the center of the Fabry-Perot interference pattern the angle of incidence, *θ*, is zero.

The convention used in this paper is that the phase shift on reflection, *ϕ*, represents an increase in optical path. The angular phase shift, *ϵ* = 2*πϕ*/λ, enters the expressions for the reflected waves as follows (*θ*=0; see fig. 1)

$$\begin{array}{l} E_{1}=(\text{wave incident on surface}\;2)\\ \;=A_{1}e^{i(\omega T-\kappa x_{2})}, \end{array}$$ (10)

$$\begin{array}{l} E_{2}=(\text{reflected wave})\\ \;=A_{2}e^{i(\omega T-\kappa x_{2}-\epsilon_{2})}, \end{array}$$ (11)

$$\begin{array}{l} E_{3}=(E_{2}\;\text{incident on surface}\;1\;\text{from inside})\\ \;=A_{2}e^{i(\omega T-\kappa x_{2}-\epsilon_{2}-\kappa t)}, \end{array}$$ (12)

$$\begin{array}{l} E_{4}=(\text{reflected component of}\;E_{3})\\ \;=A_{4}e^{i(\omega T-\kappa x_{2}-\epsilon_{2}-\epsilon_{1}-\kappa t)}, \end{array}$$ (13)

$$\begin{array}{l} E_{5}=(\text{wave}\;E_{4}\;\text{incident on surface}\;2)\\ \;=A_{4}e^{i(\omega T-\kappa x_{2}-\epsilon_{2}-\epsilon_{1}-2\kappa t)}. \end{array}$$ (14)

Here *ω* is the angular frequency, *T* the time, *κ*=2*π*/λ.

The condition for constructive interference in transmission is that the optical path difference between two successive rays should be an integral number of wavelengths.

$$\begin{array}{l} 2\pi n=\arg E_{1}-\arg E_{5}=\omega T-\kappa x_{2}-(\omega T-\kappa x_{2}-\epsilon_{2}-\epsilon_{1}-2\kappa t)\\ \;=\epsilon_{2}+\epsilon_{1}+2\kappa t\\ \;n\lambda =\phi_{2}+\phi_{1}+2t. \end{array}$$ (15)

From eqs (10) and (11) we have

$$\frac{E_{2}}{E_{1}}=\frac{A_{2}}{A_{1}}e^{-i\epsilon_{2}}.$$

For metals or for dielectric multilayers, theory can be used to calculate the quantity

$$\frac{E_{2}}{E_{1}}=\frac{A_{2}}{A_{1}}e^{i\delta_{2}}.$$

Therefore the relation between the desired angular phase shift, *ϵ*, and the calculated value, *δ*, is

ϵ=v2π−δ (16)

where *v* is an integer.

For a metal reflector the angular phase shift, *δ*, is calculated from the index of the incident medium, *μ*_0_, and the optical constants, *μ* and *k*, of the metal. The expression for the complex amplitude reflectance is [23]

$$\rho e^{i\delta }=\frac{\mu_{0}-\mu +ik}{\mu_{0}+\mu -ik}=\frac{\mu_{0}^{2}-\mu^{2}-k^{2}+i2\mu_{0}k}{\mu_{0}^{2}+\mu^{2}+k^{2}+2\mu_{0}\mu }.$$ (17)

The tangent of the phase shift *δ* is then found by taking the ratio of imaginary part to real part:

$$\tan\delta =\frac{2\mu_{0}k}{\mu_{0}^{2}-\mu^{2}-k^{2}}.$$ (18)

**Figure 2:**
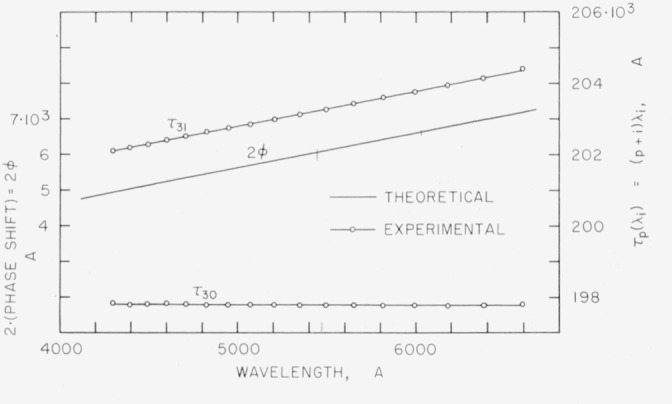
Dispersion of phase shift on reflection for aluminum films. The theoretical values were calculated from the optical constants as given by Schulz [24, 25]. For the upper experimental curve the data from table 1 and the integer *q*=31 were used. Since this curve is parallel to the theoretical curve, it follows that *q*=31 is the correct order number for the fringe at λ_0_=6593.7A. For the lower experimental curve the integer *q*=30 was used.

The quadrant for *δ* is determined by taking into account the signs of the real and imaginary parts of eq (17). Thus for $\mu^{2}+k^{2}>\mu_{0}^{2}$ (the case for all metals commonly employed in interferometry, when the separation medium is air or vacuum) the real part is negative and the imaginary part is positive, which means that *δ* is in the second quadrant. The phase shift *ϕ=ϵ*λ/2*π*=(*v*2*π−δ*)λ/2*π* therefore represents an increase of optical path of between λ/2 and 3λ/4.^3^ Figure 2 shows the phase shift for aluminum calculated from the optical constants [24, 25].

For a multilayer the phase shift can be calculated from electromagnetic theory by one of several methods. An accurate method employs multiplication of matrices, each matrix representing a single layer [26, 27]. An exhaustive treatment of the subject has been given by Abelès [28], but the outline given by Koehler [29] is adequate for many problems.

**Figure 3:**
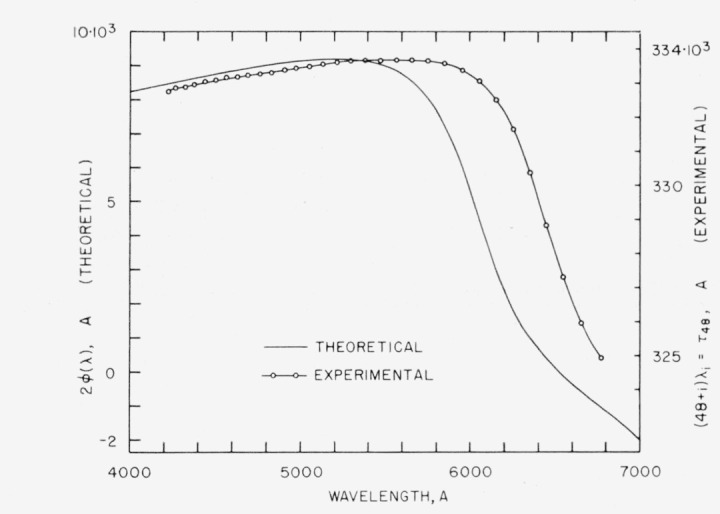
Dispersion of phase shift on reflection for 15 layer broadband reflecting films. The angular phase shift, *δ*, as given by Baumeister and Jenkins [20] was converted to linear phase shift *ϕ* by means of eq (16) in which *v*=1 and the relation *ϕ* = *ϵ*λ/2*π*. The experimental curve has the same general shape except that it is shifted to longer wavelengths. Wavelengths from table 2 were used.

In dealing with phase shifts on reflection from multilayers the concept of nodes is very useful [20]. For light reflected from a plane interface between air and a high index dielectric, the phase shift is *ϕ* = λ/2, and there is a node at the surface. For *ϕ*>λ/2, as with a metal, the node is inside the metal. For *ϕ*<λ/2, the node is on the air side of the surface. With multilayers of the common type, having a high index layer on the outside, a node for the tuned wavelength, λ, falls at the outer surface. For the broadband multilayer it has been shown [20] that there is one wavelength for which a node falls at the outer surface. At longer wavelengths the node is outside of this surface, and vice versa. Figure 3 shows the dependence of phase shift on wavelength.

## 3. Experimental Procedure

**Figure 4:**
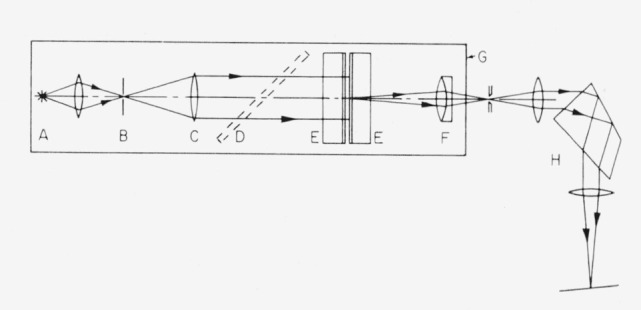
Interferometer for fringes of equal chromatic order. A. White light source. B. pinhole, C. collimator, D. beam-divider (for reflected fringes), E. flats with metal or multilayer reflecting films, F. achromatic doublet, G. interferometer base, and H. spectrograph. For Fabry-Perot interferometry the setup is changed as follows: The line source is placed at the focal point of the collimator, C. A larger spacer is introduced between the flats, E. A lens with longer focal length than F is used to focus the fringe pattern on the spectrograph slit, which in this case is wide.

**Figure 5:**
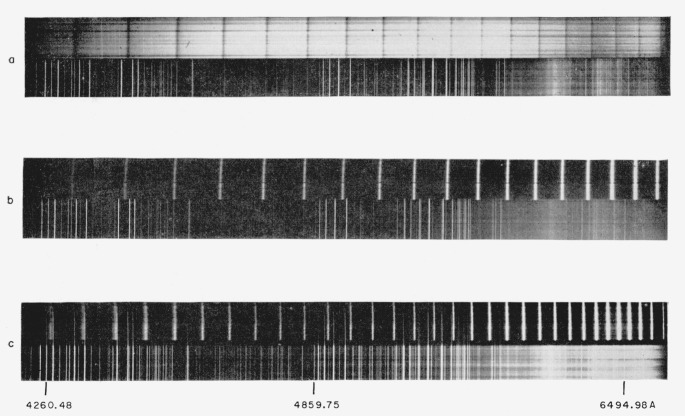
Fringes of equal chromatic order with iron reference spectra (positive print). Aluminum films, reflection fringes, 10-*μ* spacer;aluminum films, transmission fringes, 10-*μ* spacer;15 layer broadband films, transmission fringes, 17-*μ* spacer. In c, the only obvious indication of the large dispersion of phase shift is the fact that the fringe spacing does not decrease monotonically with increasing wavelength. In the red the last few fringes are more widely spaced than those immediately preceding. Where several fringes are overexposed in the red the standard deviation in determination of wavelengths is about 0.4 A, whereas in other portions of the spectrum it is from 0.1 to 0.3 A.

Data on the phase shift dispersion were obtained by means of fringes of equal chromatic order [9, 10, 11, 30]. As shown in figure 4 collimated white light illuminated the interferometer. The very small spacing, 10 to 30*μ*, was accomplished by means of aluminum foil. The transmitted light entered a spectrograph, with the image of the interferometer surface being focused on the slit. As illustrated in figure 5, bright fringes appeared in the focal plane of the spectrograph for those wavelengths at which constructive interference occurred. After photographing the fringes along with an iron arc reference spectrum, the wavelengths of the fringes were measured. The method of calculating the phase shift dispersion is given below.

Fabry-Perot interference patterns were obtained using invar spacers of 2 and 5 mm, also fused quartz spacers of 20-mm length. The light source was a natural krypton lamp, krypton being selected because of its relatively sharp and well-distributed lines. The etalons were used in air maintained near 20° C and 10-mm water vapor pressure, and corrections to the wavelengths were made by the method described by Bruce [31].

From the photograph of the interference pattern. the ring diameters were measured for several wavelengths. For the 2-mm spacer, two rings were measured at each wavelength; for the others, four rings were measured. The fractional orders, *f*, of interference at the center of the patterns were calculated with a least squares procedure described by Meissner [6].

## 4. Equations for Fringes of Equal Chromatic Order and Fabry-Perot Fringes

For the wavelengths λ_0_, λ_1_, etc., at which *feco* occur in the spectrograph, the normal incidence interference eq (15) can be written as

$$n\lambda_{0}=2t^{\prime }+2\phi (\lambda_{0}),$$ (19)

$$(n+1)\lambda_{1}=2t^{\prime }+2\phi (\lambda_{1}),\;\text{etc}.,$$ (20)

λ_0_ and λ_1_ are the wavelengths of adjacent fringes, λ_0_>λ_1_. The order number for the fringe at λ_0_ is *n*, and for each successive fringe the order number is increased by one. *t*′ is the physical separation of the reflecting surfaces, and *ϕ*(λ) is the phase shift in units of length at each surface.

In these equations the left sides can be evaluated only at the wavelengths λ_0_, λ_1_, etc., at which there are fringes. However the quantity on the right side is a continuous function of λ which is conveniently denoted as *τ_n_*(λ),

$$\tau_{n}(\lambda )\equiv 2t^{\prime }+2\phi (\lambda ).$$ (21)

The function *τ_n_*(λ) is then evaluated by plotting *n*λ_0_ versus λ_0_, (*n+*1)λ_1_ versus λ_1_, etc., and connecting the points by a smooth curve. This curve has the same shape as the 2*ϕ*(λ) curve but is displaced vertically by 2*t*′.

With the Fabry-Perot etalon the interference equations for the centers of the ring pattern at known wavelengths λ*_a_*, λ*_b_*, λ*_c_*, are:

$$(m_{a}+f_{a})\lambda_{a}=2t+2\phi (\lambda_{a}),$$ (22)

$$(m_{b}+f_{b})\lambda_{b}=2t+2\phi (\lambda_{b}),$$ (23)

$$(m_{c}+f_{c})\lambda_{c}=2t+2\phi (\lambda_{c}).$$ (24)

Here again the quantity on the right side is a continuous function of λ, the curve being displaced upward from the curve *τ_n_*(λ) by the amount 2(*t−t′*). Therefore, in general the points (*m_a_+f_a_*)λ*_a_*, (*m_b_+f_b_*)λ*_b_*, and (*m_c_+f_c_*)λ*_c_* lie along a curve which is parallel to the *τ_n_* curve.

## 5. Methods of Analysis

### 5.1. Method I

The first method for obtaining the order numbers *n* and *m* is used in cases where the phase shift dispersion is known approximately, as for example with metallic films with known optical constants for various wavelengths. For the *feco* an integer *p* is selected and the curve *τ_p_*(λ) is determined by plotting *p*λ_0_ versus λ_0_, (*p+*1)λ_1_ versus λ_1_, etc. If it does not have the same shape as the known 2*ϕ*(λ) curve, then other integers are tried until an integer, *n*, is found which makes *τ_n_*(λ) essentially parallel to 2*ϕ*(λ). This procedure is illustrated in figure 2.

Similarly for the Fabry-Perot data, integers *m_a_, m_b_, m_c_* are found such that when (*m_a_+f_a_*)λ*_a_* is plotted against λ*_a_*, (*m_b_*+*f_b_*)λ*_b_* against λ*_b_*, etc., the points fall along a curve parallel to *τ_n_*(λ). In order to measure an unknown wavelength, λ*_x_*, in terms of a standard wavelength, λ*_s_*, a modification of eq (8) is employed. Although the absolute values of *ϕ*(λ*_x_*) and *ϕ*(λ*_s_*) have not been determined, it can be seen from eq (21) that the difference, 2*ϕ*(λ*_x_*) − 2*ϕ*(λ*_s_*), is given by

$$2\phi (\lambda_{x})-2\phi (\lambda_{s})=\tau_{n}(\lambda_{x})-\tau_{n}(\lambda_{s}).$$ (25)

Therefore eq (8) becomes

$$\lambda_{x}=\frac{\left(m_{s}+f_{s})\lambda_{s}+\tau_{n}(\lambda_{x})-\tau_{n}(\lambda_{s}\right)}{m_{x}+f_{x}}.$$ (26)

The method has been described in terms of graphical procedures. A possible variation utilizes numerical calculations similar to those in the usual method of exact fractions. For wavelength λ*_a_* the value of *τ_n_*(λ*_a_*) is found from the curve or by means of a numerical interpolation of the *τ_n_*(λ) data. An integer *r_a_* is selected and the product (*r_a_*+*f_a_*)λ*_a_* is formed, also the difference (*r_a_*+*f_a_*)λ*_a_*−*τ_n_*(λ*_a_*). This is done for all the integers, *r_a_*, which lie within the range of possibility. Similarly different integers are tried for *r_b_* and *r_c_* until a set is found which satisfy the equation

$$\begin{array}{l} \left(r_{a}+f_{a})\lambda_{a}-\tau_{n}(\lambda_{a})=(r_{b}+f_{b})\lambda_{b}-\tau_{n}(\lambda_{b}\right)\\ \;=(r_{c}+f_{c})\lambda_{c}-\tau_{n}(\lambda_{c}) \end{array}$$

Once the correct integers have been found by this technique eq (26) can be used to compute unknown wavelengths.

### 5.2. Method II

The second, more general, method can be used when the phase shift dispersion is unknown. An *arbitrary* integer, *q*, is selected and the curve *τ_q_*(λ) is determined as before. Then for the Fabry-Perot data, different integers are tried for *r_a_*, *r_b_*, and *r_c_*, until a set is found for which the points (*r_a_*+*f_a_*)λ*_a_*, (*r_b_*+*f_b_*)λ*_b_*, and (*r_c_*+*f_c_*)λ*_c_* fall along a curve parallel to *τ_q_*(λ). That is,

$$\begin{array}{l} \left(r_{a}+f_{a})\lambda_{a}-\tau_{q}(\lambda_{a})=(r_{b}+f_{b})\lambda_{b}-\tau_{q}(\lambda_{b}\right)\\ \;=(r_{c}+f_{c})\lambda_{c}-\tau_{q}(\lambda_{c}) \end{array}$$ (27)

In appendix 9.1 it is shown that when the integers *r_j_* are chosen in this manner then we have

$$\left(r_{j}+f_{j})\lambda_{j}-\tau_{q}(\lambda_{j})=2(t-t^{\prime }\right)$$ (28)

regardless of the value of *q*. Here the subscript *j* represents the subscript *a*, *b, c, s*, or *x*.

The final step is to determine an unknown wavelength λ*_x_* in terms of a standard wavelength, λ*_s_*, and the measured fractions *f_x_* and *f_s_*. The integers *r_s_* and *r_x_* are determined in the same manner as *r_a_, r_b_*, and *r_c_*. When these quantities are substituted in eq (28) two equations result which can be solved for λ*_x_*:

$$\lambda_{x}=\frac{\left(r_{s}+f_{s})\lambda_{s}+\tau_{q}(\lambda_{x})-\tau_{q}(\lambda_{s}\right)}{r_{x}+f_{x}}.$$ (29)

Just as in the standard method, the wavelength λ*_x_* must be known in advance with sufficient accuracy that the proper integer, *r_x_*, can be selected. The correct integer, *r_x_*, is the one which makes the quantity (*r_x_+f_x_*)λ*_x_* fall closest to the curve (*r_j_+f_j_*)λ*_j_* versus λ*_j_*.

## 6. Results

### 6.1. Evaporated Aluminum Films

Table 1 gives the wavelengths of *feco* obtained in transmission with a 10-*μ* spacer, the aluminum films having a transmittance of 5 to 8 percent. In principle the phase shift should be calculated taking into account the finite thickness of the aluminum films [32]. However, figure 2 shows that for the thicknesses used, the phase shift values calculated from the bulk constants yield a curve quite parallel to the experimental curve. Method I was used to determine the order number, *n*, as illustrated in figure 2.

**Figure 6:**
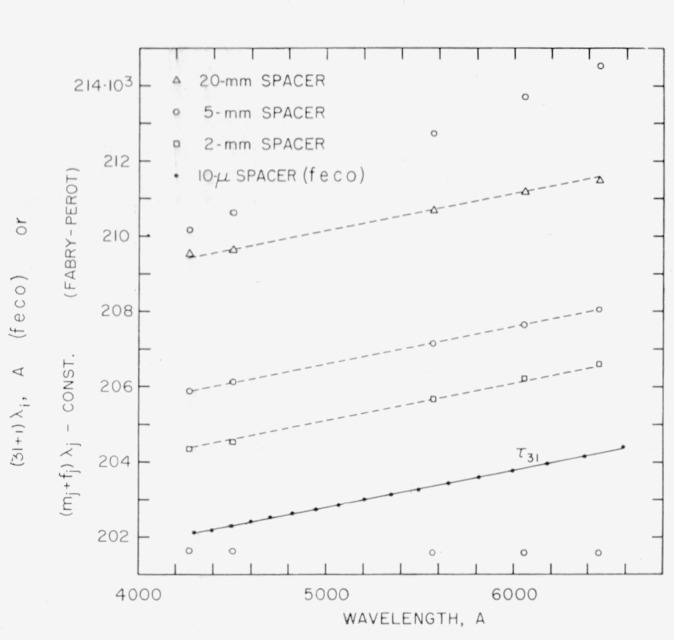
Optical path differences: aluminum films. A constant was subtracted from the (*m_i_*+*f_i_*)λ*_j_* values to bring them on the same scale as the *τ*_31_ curve obtained from transmission *feco*. For the 20-, 5-, and 2-mm spacers the constants were 401119·10^3^, 99696·10^3^, and 39691·10^3^ A respectively. For the 5-mm spacer (open circles) several different sets of integers, *m_j_*, were tried together with the appropriate fractions, *f_i_*. One set gives values of (*m_j_+f_j_*)λ*_j_* which lie along the dashed line, which is parallel to *τ*_31_. The other sets give points which obviously do not lie along lines parallel to *τ*_31_. For the 2- and 20-mm spacers the correct integers plus fractions are given in table 1, and the optical path differences are plotted above.

For the 2-, 5-, and 20-mm spacers the integers, *m*, were found as described in 5.1 and illustrated in figure 6. With each spacer the points have the same vertical displacement from the curve *τ_n_*, within experimental error.

As an example, the wavelengths 5570.34 and 4502.39 are considered being known approximately to the nearest 0.02 A, this precision being necessary to establish the order numbers. The more precise values are to be determined by interferometric comparison with the standard line at 6056 A. From figure 6,

$$\tau_{31}(5570)-\tau_{31}(6056)=203,350-203,830=-480\;A.$$

Therefore from eq (26) we have

$$\begin{array}{l} \lambda_{x}=\frac{66267.895\times 6056.1779-480}{72047.649}\\ \;=5570.3369\;A. \end{array}$$

Similarly for λ*_x_*=4502 A

$$\begin{array}{l} \lambda_{x}=\frac{66267.895\times 6056.1779-1530}{89136.737}\\ \;=4502.3931\;A. \end{array}$$

These values agree to experimental accuracy with the wavelengths for ambient conditions, as given in table 1.

In illustrating method II, rather than choose an arbitrary integer, we choose an integer, *q*, such that *τ_q_* is as nearly constant as possible. As shown in figure 2, *τ*_30_ has only a very slight negative slope. The integers, *r_j_*, which make the points (*r_j_*+*f_j_*)λ*_j_* lie along a curve parallel to *τ*_30_ are one less than the corresponding integers *m_j_*, in table 1. That is, for λ=6456, *r_j_* = 62,159.

Then for the unknown line, λ*_x_*≈4502.39 A

$$\begin{array}{l} \lambda_{x}=\frac{66266.89\times 56056.1779-197770+197,800}{89135.737}\\ \;=4502.3932\;A. \end{array}$$

Thus with aluminum films if a certain integer, *q*, is chosen the *τ_q_*(λ) curve is nearly constant and the phase shift correction is very small. Furthermore the quantities (*r_j_*+*f_j_*)λ*_j_* are nearly constant. In the standard method of exact fractions the integers are selected so that the quantities (*r_j_*+*f_j_*)λ*_j_* are equal. Therefore the method of exact fractions as normally used gives integers, *r_j_*, which are each one less than the “true” order numbers, *m_j_*.^4^ This introduces no error into the determination of unknown wavelengths because only *differences* in order numbers for two different etalon spacings are used in the final calculation.

### 6.2. Fifteen-Layer Broadband Reflectors

Table 2 gives the wavelengths of *feco* obtained in transmission for a broadband multilayer [19] made from alternate layers of cerium dioxide and magnesium fluoride [33]. The measured reflectance was between 83 and 94 percent over the region 4500 to 7000 A. In figure 3 the theoretical phase shift curve was obtained from values published by Baumeister and Jenkins [20]. The experimental phase shift curve confirms the general shape of the theoretical curve. The displacement of about 400 A to longer wavelengths indicates that the individual layers are somewhat thicker than the theoretical values [19]. This is confirmed by the location of the reflectance maximums and minimums.

**Figure 7:**
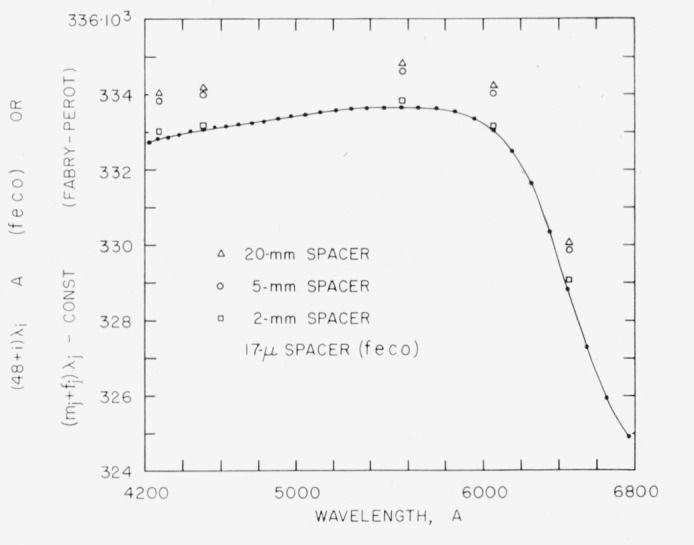
Optical path differences: 15 layer broadband films. τ_48_ was obtained from *feco* in transmission. See table 2. For the 2-, 5-, and 20mm spacers, the correct integers *m_j_* were selected as described in section 5.1. Each set of points, (*m_j_+f_j_*)λ*_j_*, lies along a curve parallel to the *τ*_48_ curve. The constants subtracted from (*m_j_*+*f_j_*)λ*_j_* values for the 20-, 5-, and 2-mm spacers were 401047·10^3^, 99552·10^3^ and 39592-10^3^A respectively.

For the 2-, 5-, and 20-mm spacers the integral orders of interference, *m*, were obtained as described in section 5.1. For each spacer, the total optical path differences (*m_j_+f_j_*)λ*_j_* plotted against λ*_j_* fell along a curve parallel to the *τ*_48_ curve, as shown in figure 7.

Table 3 gives data for the calculation of wavelengths 5570 and 4502 A, taking as the standard the line at 6056 A. Both method I and the “standard” method (eq 6) have been used.

### 6.3. Precision of Measurements

In the determination of wavelength of fringes the standard deviation for an individual reading was generally from 0.1 to 0.3 A. This resulted in a standard deviation for the values of *τ* of from 5 to 10 A, with a rare value as high as 30 A. With Δ*τ* = 10 A as an example and λ = 5000 A, the ratio 2Δ*ϕ*/λ discussed earlier in connection with eq (9) becomes 2Δ*ϕ*/λ=Δ*τ*/λ=0.002. This is to be compared with the usual error in exact fraction determination, Δ*f*=0.01.

The improvement in precision of wavelength measurement is not so dramatic, however, since this is limited by the errors in measuring the fractions *f_s_* and *f_x_*. For example, in the standard method, eq (6), we take data from the 20-mm and 5-mm spacers (table 2) on the known line and the unknown line, 5570 A. For Δ*f_s_*=Δ*f_x_*=0.01, eq (7) yields Δλ*_x_*=0.0022 A.

To compute the error obtained in the method employing *feco*, we take Δ*f_s_*=Δ*f_x_*=0.01 and 2Δϕ(λ)/λ=Δ*τ*/λ=0.002. Then eq (9) yields Δλ*_x_*=0.0012 A.

The precision of both methods can, of course, be improved by repeated readings. For example, Stanley and Meggers [34] report that in the measurement of iron lines with 20- or 25-mm spacers, the average of 5 or 6 readings had a probable error of 1 part in 7 million.

The question arises as to the best etalon spacing to be used for *feco*. Since the wavelength is multiplied by the order number to obtain the *τ* curve, smaller spacings and the resulting low order numbers tend to give smaller errors. But smaller spacings give fringes widely spaced in the spectrum. If the phase shift has a nonlinear variation with wavelength, as with the broadband multilayer, it is desirable to have the experimental points fairly close together. In this case, the best spacing was found to be 10 to 20*μ*, whereas for aluminum the smallest possible spacing was desirable.

### 6.4. Differences Between Transmission and Reflection Data

By placing a semireflecting mirror between the collimator and interferometer in figure 4 it is possible to project the light reflected from the interferometer onto the spectograph slit. With a small separation between the interferometer plates the resulting spectrum consists of dark fringes crossing the bright continuum. See figure 5a.

If the reflecting surfaces are nonabsorbing, the principle of conservation of energy requires that the reflected spectrum be the exact complement of the transmitted spectrum. Under these circumstances the wavelengths of the fringes should be the same in the two spectra. Then the *τ_n_* curve plotted from the reflection data should also give the phase shift dispersion.

If the reflecting films are partially absorbing, this conclusion does not necessarily hold. Holden [35] has shown that there can be asymmetry in the intensity distribution of fringes obtained in reflection.

**Figure 8:**
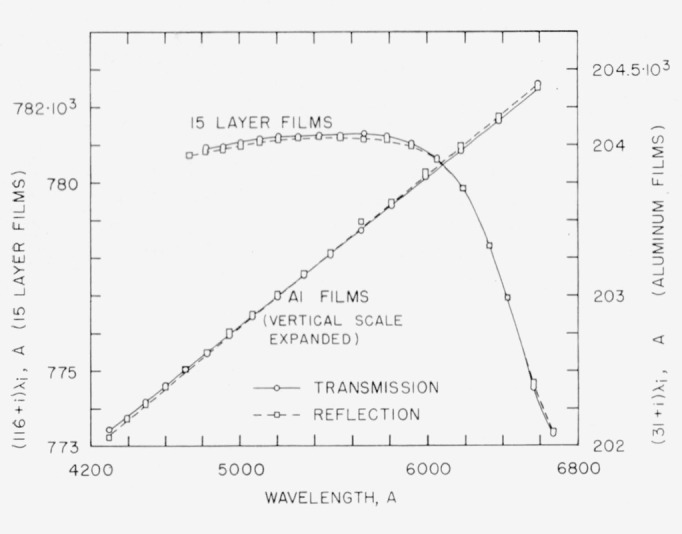
Differences in data obtained by transmission and reflection. The experimental points represent (*n+i*)λ*_i_*, where *n* is the order number of the fringe at λ_0_, *i* is an integer, and λ*_i_* is the wavelength of a fringe observed either by transmission or by reflection. In the case of the 15-layer films a relatively thick spacer (40*μ*) was used and fringes were closely spaced. Only about one-third of the fringes were measured and plotted. In the case of the aluminum films the vertical scale interval has been expanded to four times that of the multilayer in order to show more clearly the small but significant difference between the transmission and reflection data. Only the transmission *feco* should be used in connection with Fabry-Perot fringes obtained by transmission.

Experiments were conducted to determine whether or not the *τ_n_* curve obtained from reflection *feco* was the same as that obtained by transmission. Exposures were taken within a few minutes of each other, and the same areas of the reflecting films were used. As expected, the aluminum films showed a small but significant difference. As shown in figure 8 the slopes are different, the maximum discrepancy between 4200 and 6500 A amounting to about 40 A. The reflection fringes showed a definite asymmetry, which made determination of their wavelengths more difficult.

The two curves for the 15-layer multilayer showed a difference of about 150 A between 4800 and 6600 A. See figure 8. This result was unexpected because of the low absorption of dielectric multilayers. Transmittance and reflectance measurments on these multilayers indicated with the absorptance is no more than 2.5 percent at 4800 A and no more than 1 percent at 6000 A. However, no error is introduced if phase shift data obtained by transmission *feco* are used in connection with Fabry-Perot fringes obtained in transmission since both are governed by the interference eq (15). The reflection fringes are governed by a different interference condition [11, 35, 36].

### 6.5. Uniformity of Phase Shift Dispersion

With multilayer films the reflectance and phase shift on reflection are generally more sensitive functions of the thickness of the individual layers than with metal reflectors. If the absolute value of the phase shift is not constant over the aperture, it has the same effect as nonflatness of the support plate. If the *dispersion* of phase shift is not constant, then the shape of the reflected wavefront will depend on the wavelength [37]. In particular, if two such mirrors are adjusted to be optically parallel when a green line is used, they may not be optically parallel in the red or blue.

The phase shift dispersion was measured at six points across the 15-layer films. Over a 40-mm distance there was a definite change in the shape of the curve, amounting to 300 A from the blue to the red end of the spectrum. For precision Fabry-Perot interferometry this variation is too large to be tolerated. For the experiments reported here a 10-mm-diam aperture was placed between the interferometer plates. The total variation in phase shift dispersion over this area was less than 100 A, which is the same order of magnitude as the departure from flatness of the support plates.

Freshly deposited aluminum films showed very little variation in phase shift dispersion over a 40-mm aperture, the maximum discrepancy being about 40 A over the region 4300 to 6600 A.

These results indicate that multilayers for interferometers in which several wavelengths are to be used should be checked for uniformity of phase shift dispersion. The use of *feco* provides a convenient and precise method.

## 7. Conclusions

It has been found that measurements of reflection phase shift dispersion by means of fringes of equal chromatic order are useful at three stages in wavelength comparisons by Fabry-Perot interferometry. First, for multilayer reflectors it must be established that the phase shift dispersion is sufficiently uniform over the aperture. This can be done by obtaining the *feco* at several points across the aperture.

Second, the phase shift data can be combined with the method of exact fractions to yield the integral order numbers. This is especially valuable in the case of multilayers where the dispersion of the phase shift is high and where calculated phase shifts may be in error due to poorly known layer thicknesses.

Third, the accuracy of measurements of unknown wavelengths can be improved somewhat. The basic reason is that for high reflection mirrors, wavelengths of *feco* can be measured to about ±0.002 order, whereas the error in determining fractions in Fabry-Perot patterns is about ±0.01 order. This difference is due primarily to the fact that *feco* are obtained from a very small area of the plate. Small departures from flatness of the substrate surface do not reduce the sharpness of *the feco*. On the other hand, the Fabry-Perot fringes result from interference over the entire aperture, and variations in the surface reduce the fringe sharpness. In return for the gain in precision afforded by the *feco* the necessary precaution is that the variation in phase shift dispersion across the aperture is sufficiently small.

## Figures and Tables

**Table 1:** Fabry-Perot and feco data obtained with aluminum films

Ambient conditions for Fabry-Perot data, 20-mm, 5-mm spacers: 21.2°C, 754.6mm Hg barometric pressure, 10-mm vapor pressure; 2-mm spacer: 22.0°C, 758.8-mm, 10-mm vapor pressure.		
Wavelengths of *feco* λ*_i_*	*n+i*	Optical path differences (*n*+*i*)λ*_i_*
		
*A*		*A*
6593.7	31	204, 404
6379.8	32	204,153
6180.7	33	203,962
5993.7	34	203, 787
5817.1	35	203, 599
5650.9	36	203,433
5493.8	37	203,272
5345.5	38	203,129
5205.1	39	202, 998
5071.6	40	202, 862
4944.7	41	202, 734
4824.2	42	202, 616
4709.5	43	202, 510
4600.1	44	202,404
4495.5	45	202, 295
4395.4	46	202, 191
4300.3	47	202,112

Wavelength of krypton line, λ*_j_* (ambient)	Order, *m*, of interference plus fraction, *f*	Optical path difference, (*m+f*)λ*_i_*

20-mm spacer		

*A*		*A*
6456.3448	62160.634	401, 330, 486
6056.1779	66267.895	401,330,161
5570.3369	72047.649	401, 329, 678
4502.3929	89136.737	401, 328, 612
4274.0066	93899.833	401, 328, 506

5-mm spacer		

6456.3448	15473.779	99, 904,053
6056.1779	16496.155	99, 903, 649
5570.3369	17934.851	99, 903,162
4502.3929	22188.669	99, 902, 106
4274.0066	23374.296	99, 901, 895

2-mm spacer		

6456.3399	6179.600	39, 897, 598
6056.1733	6587.857	39, 897, 204
5570.3326	7162.346	39, 896, 649
4502.3894	8860.985	39, 895, 605
4274.0033	9334.420	39, 895, 342

**Table 2:** Fabry-Perot and feco data obtained with 15-layer broadband multilayers

Ambient conditions for Fabry-Perot data: 19.5° C, 757.05 mm Hg barometrie pressure, 6.5-mm vapor pressure.		
Wavelengths of *feco* λ*_i_*	*n+i*	Optical path difference (*n+i*)λ*_i_*
		
*A*		*A*
6769.1	48	324,920
6651.8	49	325,937
6447.2	51	328,807
6257.5	53	331,647
6055.4	55	333,049
5852.1	57	333,571
5655.3	59	333,600
5469.9	61	333,661
5295.6	63	333,623
5131.5	65	333, 548
4976.4	67	333,421
4830.4	69	333,295
4693.1	71	333,209
4563.4	73	333,128
4440.2	75	333,014
4323.3	77	332,891
4211.6	79	332,713

Wavelength of krypton line, λ*_j_* (ambient)	Order, *m*, of interference plus fraction, *f*	Optical path difference, (*m+f*)λ*_j_*

20-mm spacer		
*A*		*A*
6456.3278	62168.014	401,377,077
6056.1620	66276.498	401, 381, 209
5570.3222	72057.199	401,381,815
4502.3809	89148.651	401, 381,184
4273.9952	93912.374	401, 381,036

5-mm spacer		

6456.3278	15470.386	99, 881,883
6056.1620	16493.291	99, 886,042
5570.3222	17931.927	99, 886, 611
4502.3809	22185.150	99, 885, 996
4273.9952	23370.598	99, 885, 824

2-mm spacer		

6456.3278	6183.25	39, 921, 087
6056.1620	6592.49	39, 925,189
5570.3222	7167.60	39, 925, 841
4502.3809	8867.57	39, 925,178
4273.9952	9341.38	39, 925,013

**Table 3:** Wavelength calculations from Fabry-Perot and feco data obtained with multilayer reflectors

a. Calculation using eq (26), 20-mm spacer and *feco*.			
λ*_s_*	(*m_s_*+*f_s_*)	(*m_s_*+*f_s_*)λ*_s_*	*τ*_48_(λ*_s_*)
			
*A*		*A*	*A*
6056.1620*	66276.498	401,381,209	333, 030

λ*_x_*†	*τ*_48_(λ*_x_*)	(*m_x_+f_x_*)	λ*_x_*	λ*_x_*‡
				
*A*	*A*		*A*	*A*
5570.32	333, 650	72057.199	*5570.3224	5570.3222
4502.38	333,070	89148.651	*4502.3816	4502.3809

b. Calculation using eq (6), 20-mm and 5-mm spacers.			
λ*_x_*†	(*m_s_*′+*f_s_*′)	(*m_x_*′+*f_x_*′)	λ*_x_*
			
*A*			*A*
5570.32	16493.291	17931.927	*5570.3215
4502.38	16493.291	22185.150	*4502.3806

* The wavelengths of the standard line at 6056 A and of the unknown lines are for the ambient conditions given in table 2.

† Rough value.

‡ From Bruce [31] (converted to ambient conditions).

## Footnotes

2 Figures in brackets indicate the literature references on page. 199.

3 The integer *v*=1 was selected for simplicity. Some other authors select *v*=0, which makes *ϕ* represent a *decrease* of optical path of between λ/4 and λ/2. For an infinite wave train the two interpretations are physically identical. They do lead to different order numbers of interference.

4 The word “true” is in quotation marks because some authors use another convention for sign of the phase shift, which results in another order number. See section 2.

## 9. Appendix

### 9.1. Derivation of Equation (28)

It is to be shown that for an arbitrary integer *q* and for integers, *r_j_*, selected as described, the differences(*r_j_+f_j_*) λ*_j_*−*τ_q_* (λ*_j_*) are equal to 2 (*t−t′*). To both sides of eq (19) we add the quantity (*q−n*) λ_0_.

$$q\lambda_{0}=2t^{\prime }+2\phi (\lambda_{0})+(q-n)\lambda_{0}.$$

Adding (*q−n*) λ_1_ to each side of eq (20) gives

$$(q+1)\lambda_{1}=2t^{\prime }+2\phi (\lambda_{1})+(q-n)\lambda_{1}.$$

Therefore the smooth curve connecting the points *q*λ_0_, (*q*+1) λ_1_, etc., is the continuous function

$$\tau_{q}(\lambda )\equiv 2t^{\prime }+2\phi (\lambda )+(q-n)\lambda .$$ (30)

Now from eq (22) we obtain the relation

$$\begin{array}{l} (r_{a}+f_{a})\lambda_{a}=(m_{a}+f_{a})\lambda_{a}+(r_{a}-m_{a})\lambda_{a}\\ \;=2t+2\phi (\lambda_{a})+(r_{a}-m_{a})\lambda_{a} \end{array}$$ (31)

The integers *r_a_, r_b_*, etc., are selected so as to satisfy eq (27). Substitution of eq (31) and the corresponding equation for λ*_b_* into eq (27) yields

$$\begin{array}{l} (r_{a}+f_{a})\lambda_{a}-\tau_{q}(\lambda_{a})=2t+2\phi (\lambda_{a})+(r_{a}-m_{a})\lambda_{a}\\ -[2t^{\prime }+2\phi (\lambda_{a})+(q-n)\lambda_{a}]=2t+2\phi (\lambda_{b})+(r_{b}-m_{b})\lambda_{b}\\ -[2t^{\prime }+2\phi (\lambda_{b})+(q-n)\lambda_{b}] \end{array}$$

or

$$(r_{a}-m_{a}-q+n)\lambda_{a}=(r_{b}-m_{b}-q+n)\lambda_{b}.$$ (32)

Since *r*, *m*, *q*, and *n* are integers, the only general way in which eq (32) can be satisfied is *r_a_−m_a_ =r_b_−m_b_=q−n*, in which case

$$\left(r_{j}+f_{j})\lambda_{j}-\tau_{q}(\lambda_{j})=2(t-t^{\prime }\right)$$ (33)

where *j* stands for *a*, *b*, or *c*. Thus the difference between the etalon spacings can be determined, regardless of the phase shift dispersion and of the integer, *q*, which is arbitrarily selected.

### 9.2. Required Accuracy of Theoretical Phase Shift Curve

The phase shift curve must be known from theoretical calculations with a certain accuracy if the “true” order numbers are to be determined, as in section 5.1. We wish to show that the slope of the curve 2*ϕ* versus λ must be known to better than ±0.5.

With *feco* the criterion for selection of the true order number, *n*, for the fringe at λ_0_ is that the plot of *n*λ_0_ versus λ_0_, (*n+*1)λ_1_ versus λ_1_, etc., gives a curve *τ_n_*(λ) which is parallel to 2*ϕ*(λ), i.e., *τ_n_*(λ)=2*t*′+2*ϕ* (λ). The curve which results for an incorrect integer, *q*, is given by eq (30). Now the smallest possible error is one unit, i.e., *q=n+*1. Then by eq (30),

$$\tau_{n+1}(\lambda )=2t^{\prime }+2\phi (\lambda )+\lambda .$$

It is evident that at all values of λ the slope of the *τ_n_*_+1_(λ) curve is greater by one than the slope of the *τ_n_*(λ) curve. Thus the possible *τ_q_* curves are a family of curves which at a given value of λ differ in slope by one. In order to make the correct selection of *n*, therefore, the slope of the 2*ϕ*(λ) curve should be known to better than ±0.5.

Similarly for Fabry-Perot data, the criterion for selection of the order numbers *m_j_*(*j=a,b*, or *c*) is that when the values of (*m_j_*+*f_j_*)λ*_j_* are plotted against λ*_j_* the points fall along a curve, *A*, parallel to 2*ϕ*(λ). If the integers, *m_j_*, are each increased by one, the resulting points (*m_j_+*1+*f_j_*)λ*_j_* versus λ*_j_* fall along a curve having a slope greater by one than curve *A*. Therefore in order to make the correct selection of *m_j_*, the slope of the 2*ϕ*(λ) curve should be known to better than ±0.5.

This is not a severe restriction, amounting to a maximum permissible uncertainty in *ϵ*=27*πϕ*/λ of about ±35° over the wavelength interval between 5000 and 6000 A. Therefore in most cases of metallic reflectors and multilayers the theoretical phase shift will be known accurately enough to calculate the true order numbers. When it is not known with sufficient accuracy, method II can be used.
